# K-wire fixation vs 23-gauge percutaneous hand- crossed hypodermic needle for the treatment of distal phalangeal fractures

**DOI:** 10.1186/s12891-020-03606-6

**Published:** 2020-09-02

**Authors:** Letizia Senesi, Andrea Marchesini, Pier Paolo Pangrazi, Marialuisa De Francesco, Antonio Gigante, Michele Riccio, Francesco De Francesco

**Affiliations:** 1 Department of Reconstructive Surgery and Hand surgery, Azienda Ospedaliera Universitaria “Ospedali Riuniti”, Via Conca 71, 60126 Ancona, Italy; 2 Clinical Orthopaedics, Polytechnic University of Marche, Ancona, Italy; 3 Data Analysis Office, University of Milan, Milan, Italy

**Keywords:** Distal interphalangeal fracture, Needle fixation, K-wire fixation, Range of motion, Time to union

## Abstract

**Background:**

Distal Phalanx (DP) fractures are the most common hand injuries. Bone fixation associated with soft tissue reconstruction, is often required to ensure more effective outcomes. The aim of the present study is to compare functional outcomes of DP fractures surgically treated with crossed manual drilled 23 Gauge needles vs crossed Kirschner-wires (k-wire).

**Methods:**

Clinical data included analysis of patient demographics, range of motion (ROM), and complications. Radiographic assessment considered fracture type, location, fracture displacement, and radiographic union. Functional outcomes analysis was performed.The statistical significance was assessed at the level of probability lower than 5%.

**Results:**

A total of 60 patients from 2012 to 2015 were retrospectively enrolled and among them 12 patients suffering from diabetes or current smokers. A total of 60 DP fractures were treated, 32 with needles (group A) and 28 with k-wire fixation (group B). Time to union, showed in different time points, was significantly lower in group A (≤ 40 days, *p* = 0.023*) compared to group B. ROM of the distal interphalangeal joint at six months follow-up was 60° in group A and 40° in group B. A significant improvement was observed (*p* = 0.001*) in the 23 G needle treated group. Functional outcome analysis showed that VAS was significantly lower in group A compared to group B (*p* = 0.023*).

**Conclusion:**

Our study showed that the 23 G needle yielded satisfactory results in terms of time to union and range of motion compared to k-wire fixation especially for tuft and shaft DP fractures. Therefore, should be a valid alternative to k-wire fixation in selected patients.

## Background

Hand fractures are among the most frequent skeletal injuries. In particular, the most commonly fractured bone in the hand is the distal phalanx (DP) [1]. These fractures mostly occur in young workers of either sex, as a result ofcrush and avulsion events.

Soft tissue trauma is a constant characteristic of these fractures. As a consequence, treatment is oftenoriented towards the nail-bed and pulp tissue as compared to the fracture itself [2].

The nail provides stability, sustains the soft tissue of the fingertip and aids an important functions such as delicate movements andlifting of tiny objects with the pincer function [3]. The repositioning of the fingernail contributes to a form of external osteosynthesis.

Fracture treatmentusually involves a period of protective splinting until pain resolution and healing of fracture [4].

DaCruz et al. [5] approximately 30 years ago, had reporteddrawbacks inconservative treatment with high rates of residual disability such as dysesthesia, onychodystrophy, loss of Range of Motion (ROM), pain as well as fracture instability.

Several studies [6, 7] outlined the symptomatic nonunion of DP fractures defining hence, a need for stable bone fixation to avoid undesired adverse effects.

In literature, different techniques of internal fixation have beendescribed: some authors recommended a 20-gauge hypodermic needle drilled across the fractures [2], others proposed Kirschner-wires (k-wire) fixation [8] and the cortical screw of 1.3–1.5 mm [9–11] drilled across the fracture was considered a promising technique.

The k-wire fixation is the most frequently used technique, but complications are reported in literature such as stiffness, pin site infection and fracture instability [12].

Herein, we report the use of a technique inspired by Cheng et Schneider for the treatment of DP fractures.

The purpose of this study is to investigate the operative fixation for DP fractures using two 23-Gauge crossed hypodermic needle hand-drilled into the fractures compared tocrossedk-wire, proposing these two methods in skeletally adult patients in relation to fracture union and functional outcome.

## Methods

We performed a retrospective study of patients sufferingfrom DP fractures, treated in our hospital from 2012 to 2015. This study was approved by a local ethics committee(OR567–19). All procedures involving human participants were in accordancewith the ethical standards of the institutional and national research committee and with the 1964 Helsinki declaration and its later amendments or comparable ethical standards.

Our inclusion criteria comprised patients with solitary DP fractures subjected to surgical fixation with either two hand-crossed 23-G hypodermic needle or two crossed k-wire.

Clinical data included patient demographic data, mechanism of trauma, type of treatment, follow-up, range of motion (ROM), complications, final functional outcomes.

Radiographic analysis included fracture type (transverse, comminuted), location (tuft, shaft and articular) and radiographic union.

Patients with diabetes and smoking history were included in this study. We excluded patients with mallet finger and concomitant fractures of the same handin order to reduce confounding factors.

Patients were divided into two groups based on methods of fixation (Group A: 23-Gauge needles; Group B: k-wire).

### Treatment details

All treatments were performed under local anesthesia. Following fracture reduction, in Group A, fractures were treated usingthe two hand-drilled, crossed 23-G hypodermic needle. In Group B, fractures were treated using twocrossed 0.8–1 mm k-wire, drilled from the tip.

Patients belonging to group A, were treated in a surgical room in the emergency department (ED), (without the physical presence of anesthesiologist an nursing staff). Those belonging to group B, underwent the treatment in the operatory room.

One or the other treatment was depending on the availability of operatory room at the moment of the trauma, and patient’s compliance.

All the patient underwent fixation with fluoroscopic control intraoperatively and postoperatively, in both groups. The patients underwent to needle fixation obtain a x-ray also in the ED. We perform the fixations until the placement of hardware were perfect.

The wire and the needle were not buried. Correct bone fixation was monitored followed by closure of needle tubeswith a needle holderin group A. All the treatments were performed by the twosurgeons with high expertise in our department.

For articular fractures, a temporary arthrodesis of Distal Interphalangeal (DIP) joint with was performed, pushing forward k-wire or needles until the head of the middle phalanx. The temporary arthrodesis was maintained 1 month, then the devices were pull back to allow DIP joint movements.

In tuft and shaft fractures DIP joint was not locked and the patient was able to move the DIP joint.

We applied a short cast on the finger to avoid wire relocation. Patients were advised to remove the cast and to change the finger dressing daily or three times a week upon cutaneous stiches removal, afterwords one or two times a week. The aim is to prevent infection and aid early mobilizationof PIP joint in all type of fractures and of DIP joint in tuft and shaft fractures. Patients were accurately advised, how to perform dressing to avoid k-wire and needles relocation.

Clinic and radiographic controls were performed on an average of 10 days and 30 (+/− 5) days after fixation. In case of absence inbone unionat30 days, X-rays were repeated after 10 days (from 30 to 70 days). Final time point for statistical analysis was fixed at 40 days. Bone union was retrospectively established by a surgeon blinded to the time point of x-rays evaluation.

### Statistical analysis

Variables were tested for normality by means of Shapiro test. A non-parametric approach was chosen for the analysis as variables were not found to be normally distributed. Qualitative variables were summarized using absolute and percentage frequencies and comparisons between the two groups were performed by means of Chi-squared test or Fisher exact test (when the expected frequencies were lower than 5). Quantitative variables were expressed as median and interquartile range (1st; 3rd quartiles) and groups were compared using Wilcoxon signed rank test for independent samples.

The statistical significance was assessed at the level of probability lower than 5%. All the analysis were performed using R version 3.5.3.

## Results

### Demographic data

From 2012 to 2015, a total of 60 patients with surgical DP fractures were treated in our clinic. Of these, 12 patients suffered from diabetes mellitus or were current smokers and were includedin the studyto reduce biases.

Of all 60 patients, 17 were female and 43 were male. The average age was 44 years for male patients and 43 for female. The most common mechanism of injury was crushing in 87% (52 patients) and cutting in 13% (8 patients).

Thirty-two patients had received 23-G crossed needle fixation (Group A) and 28 had receivedk-wire fixation (Group B). Patients with the same lesion were subjected to needle or k-wire fixationrandomly depending on emergency room or operatory room availability and patients’ compliance.

In Group A the left hand 60% (18/32 cases) was the most common site of fracture, in Group B60.9% (17/28 cases) the right hand (Table 1).

**Table 1:**
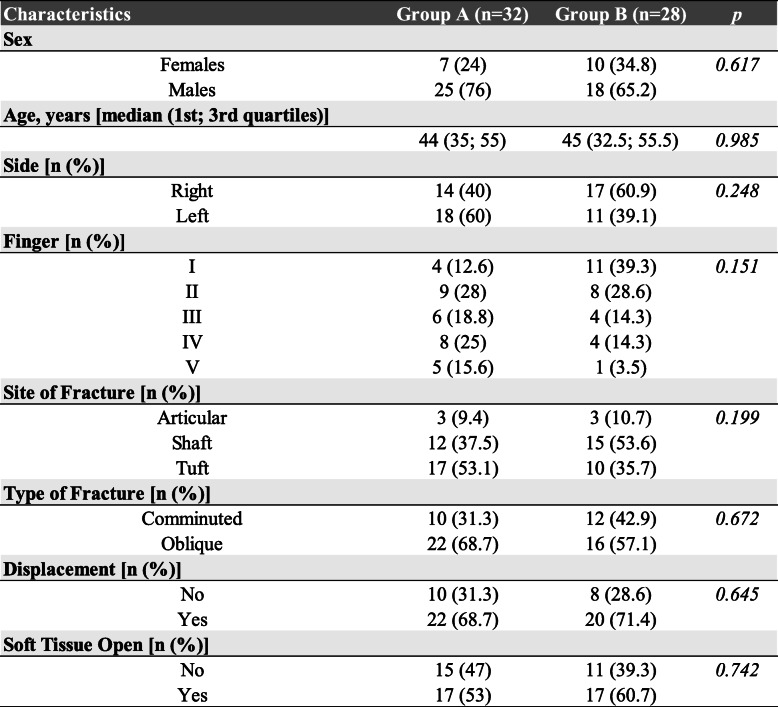
Comparison of fractures characteristics between the fixation groups

The average time of surgery was 40 min for k-wire and 26 min for 23-G needle fixation.

Needle fixation was performed in a small surgery room in the emergency department of our hospital, without the presence of nursing staff, while k-wire surgery was performed in the operatory room.

### Fracture characteristics

Transverse fracture was the most common (63% of cases, 38 patients) compared to comminuted fractures (37%, 22 patients). The most common fracture locationwere Shaft 45% (27/60), and Tuft 45% (27/60), followed by Articular 10% (6/60). Differences in fracture distribution in the two groups were not statistically significant (*p* = 0.199) (Table 1).

There was no significant difference in soft tissue injury between the twogroups of fractures and nail bed injuries (*p* = 0.74 and 0.88 respectively) Table 1.

### Time to union

**Fig. 1:**
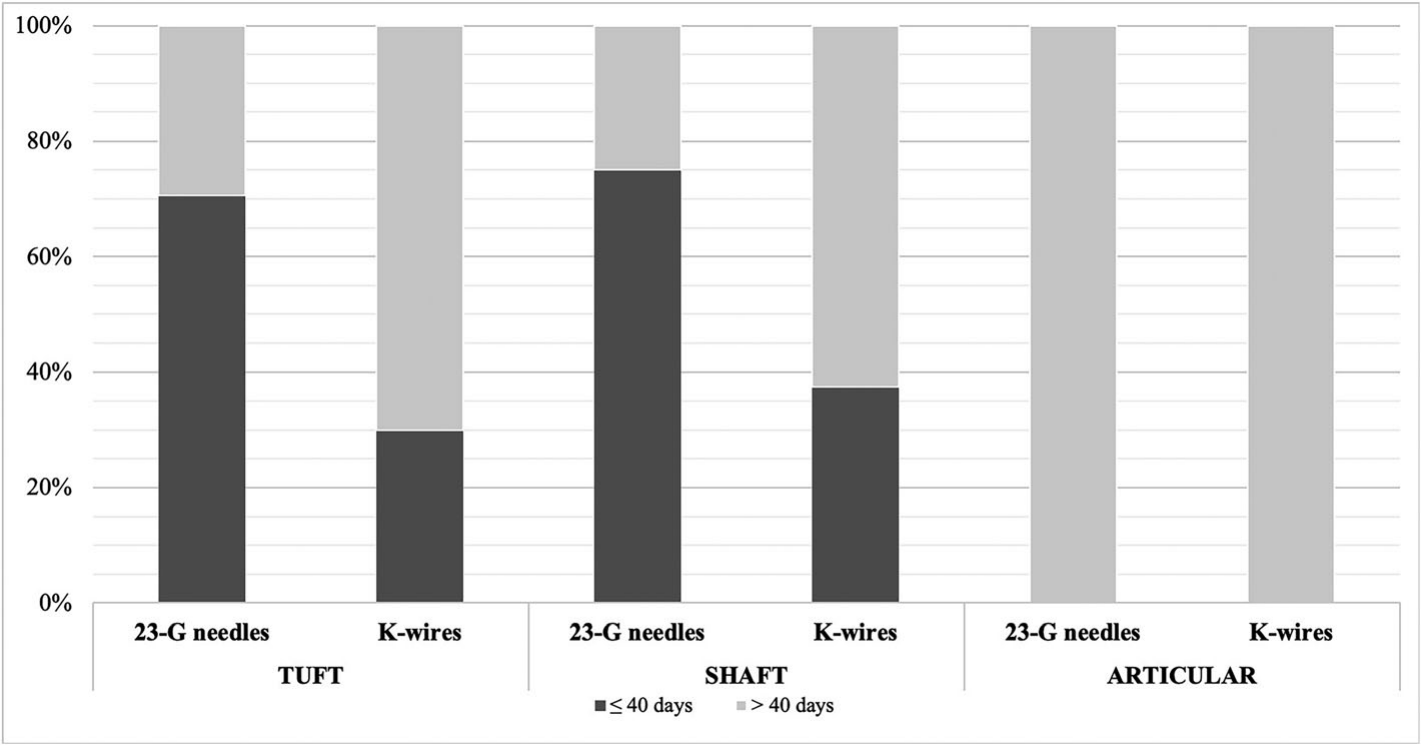
Proportion of fracture healing depending on fixation method and type of fracture. Tuft and Shaft fracture treated by needle fixation had faster healing (≤ 40 days) compared with k-wire fixation (p 0.021*)

**Fig. 2:**
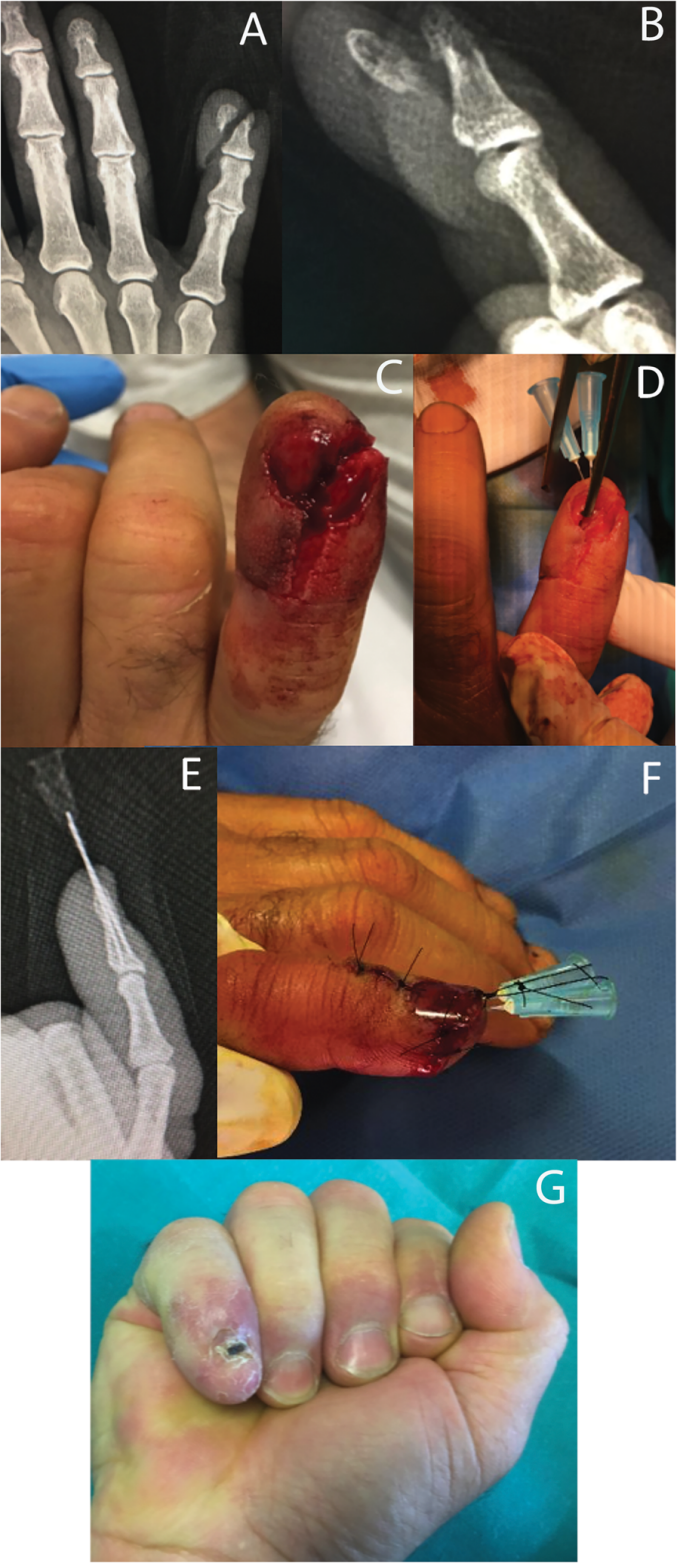
Male patient, 44 years old, with open injury of the distal phalanx and tuft fracture of the little finger (**a**,**b**,**c**). Clinical presentation after fixation of bone fragments with the hand crossed 23-G needles. The nail bed was also restored and a silicon lamina was used to protect it (**d**,**f**). X-rays performed 30 postoperatively (**e**), and clinical outcome after the removal of fixation devices (**g**)

**Fig. 3:**
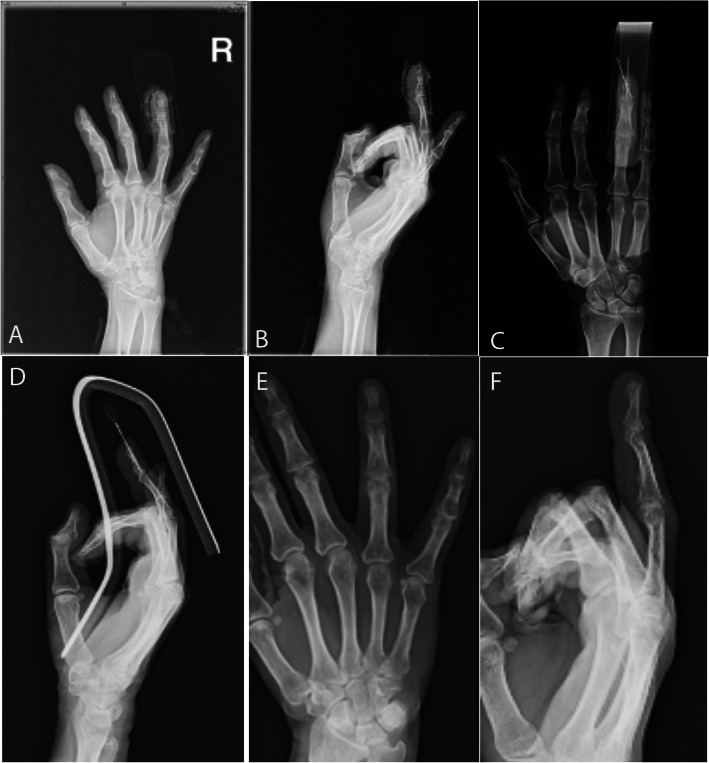
Male patient, 40 years old with crush injury and tuft fracture of the ring finger (**a**,**b**). Fixation was performed by two crossed 23-G needles (**c**,**d**). X rays after hardware removal 30 days postoperatively depict fracture union (**e**,**f**)

**Fig. 4:**
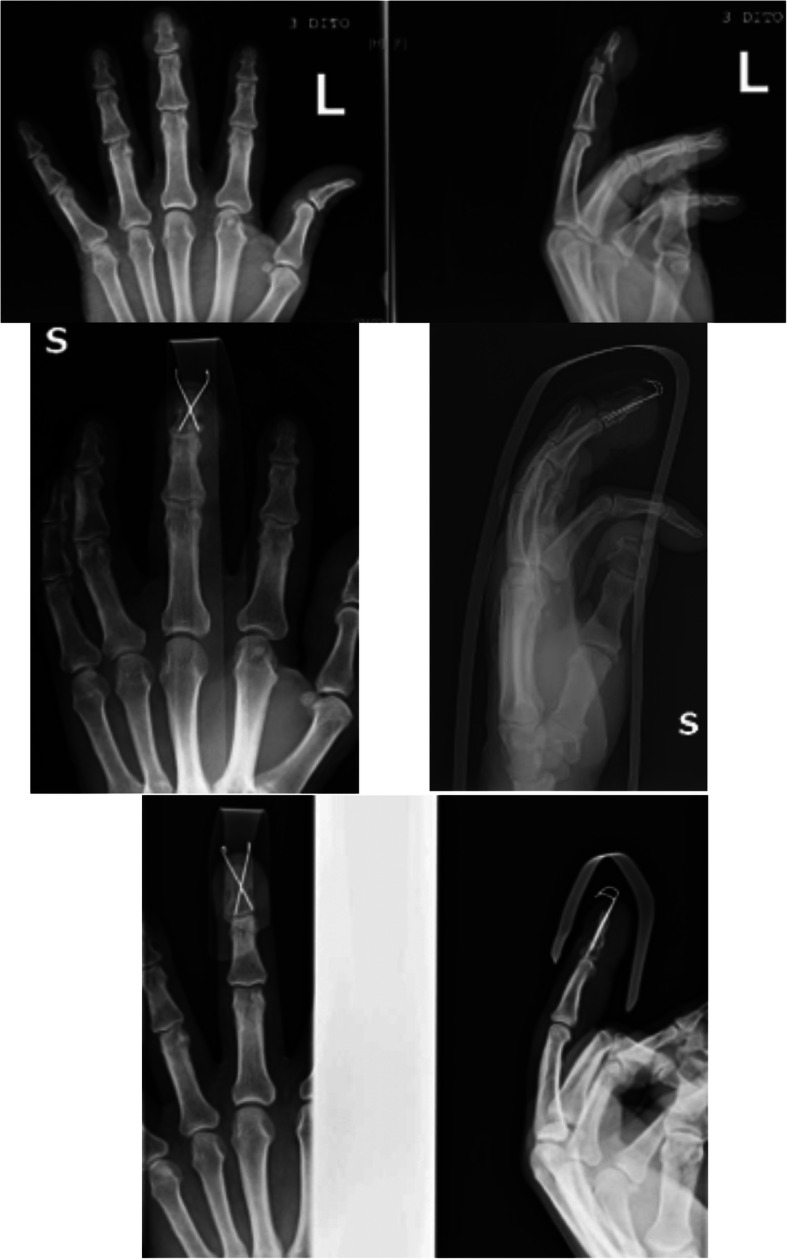
Male patient, 50 years old with crush injury and fracture dislocation of the shaft of the middle finger and dorsal soft tissue lesion. Crossed 0.8 mm k-wires fixation was performed. X-rays 50 days postoperatively depict fracture union

**Fig. 5:**
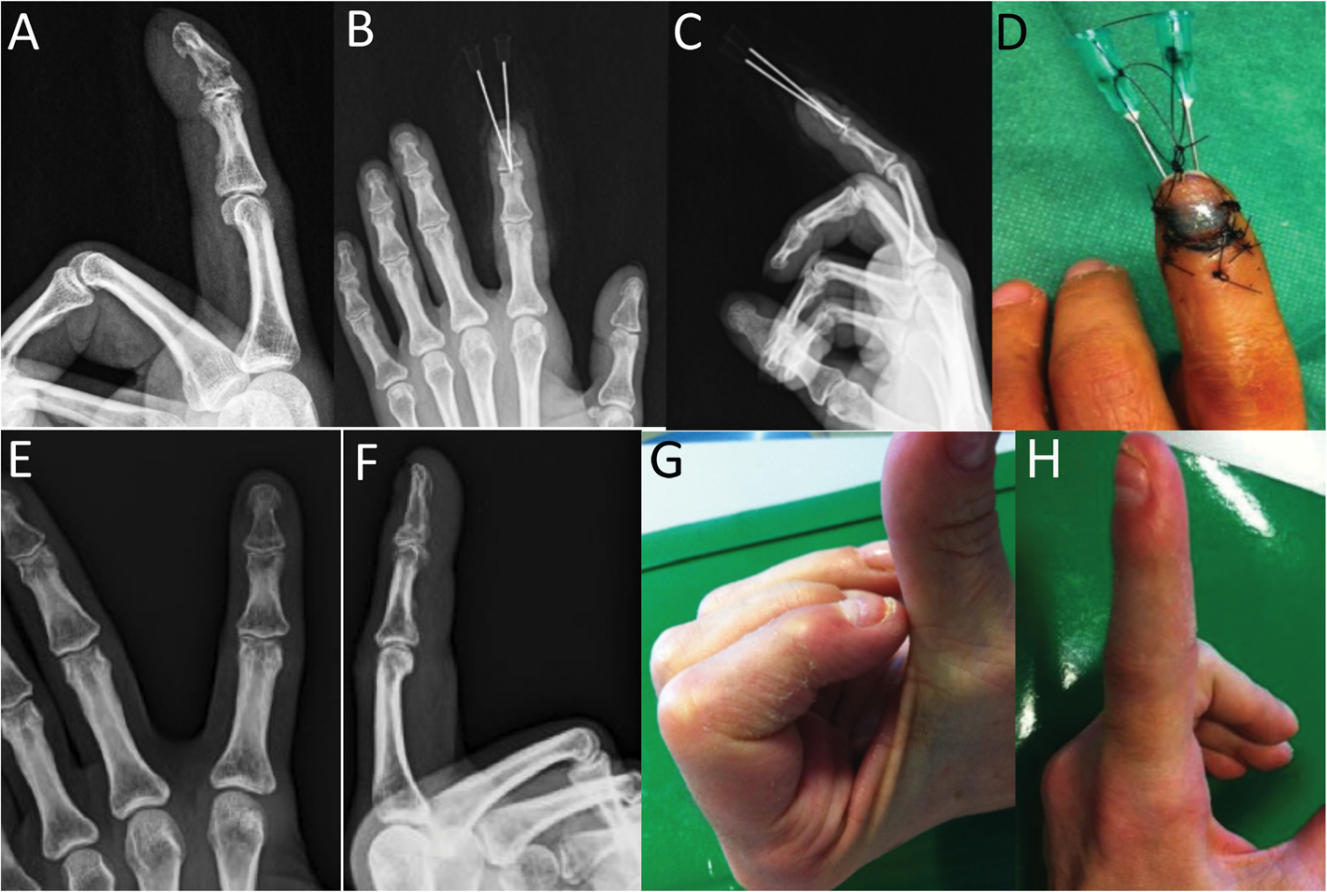
Female patient, 43 years old with crush injury of the tip of index finger and a shaft fracture (**a**) that was treated with 2 hand-crossed needles (**b**,**c**,**d**). Radiographic outcome after hardware removal 35 days postoperatively (**e**,**f**), and clinical outcome six months postoperatively (**g**,**h**)

Time to union was evaluated in several time points (Table 2 and Fig. 1) in both groups of patients. It was also fixed a time point at 40 days, statistical analysis revealed that union was significantly faster in group A (≤40 days), compared to group B (≥40 days) specifically for tuft and shaft fractures *p* value 0.023* (Figs. 1, 2, 3, 4, 5). For articular fractures, time to union in group A compared to group B revealed no statistical difference in healing (*p* = 0.1).

**Table 2:**
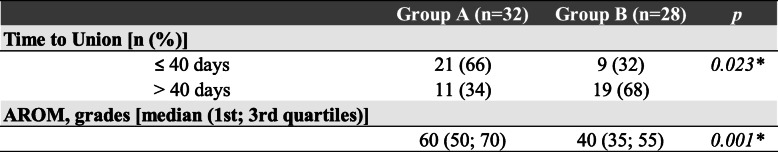
Time to union (≤ 40 days or > 40 days) and AROM six months postoperatively

No statistically significant difference (*p* = 0.9) was found between smoker/diabetic patients and the remaining patients concerning time to union, in both groups.

The differences between smoker/diabetic patients concerning time to unionin both groups were performed (Chi squared test was used at *p*-value 0.05). The differences were not statistically significant (*p* = 0.9).

### Range of motion (ROM)

Mean DIP joint ROM at the final follow-up (6 months after surgery) rounded up or downwas 60° for Group A and 40° for Group B. This difference was statistically significant (*p* = 0.001*) Table 2. We also analyzed the differences concerning ROM between the smoker/diabetic subgroup and the remaining patients. Wilcoxon test was performed at *p* value 0.05. The differences were not statistically significant (Group A: *p* = 0.29; Group B: *p* = 0.61).

### Sensibility and onychodystrophy

Six months aftersurgery, we performed the Semmes-Wenstein test and the Dellon test in both groupsof patients. We did not observe significant differences (*p* = 0.863; *p* = 0.395 respectively). No difference was reported regarding onychodystrophy in both the treated groups (*p* = 0.896) (Table 3).

**Table 3:**
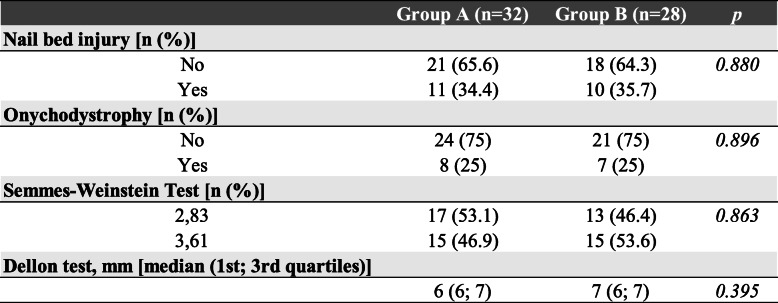
Six months postoperative clinical outcome

### Functional outcomes

Functional assessment tests were performed 6 months after surgery. Quick DASH and Pinch test (dynamometric grip strength) reveal no statistically significant differences between the groups (*p* = 0.072; *p* = 0.103 respectively). Moreover, VAS value was statistically lower in group A, compared to group B (*p* = 0.023*) Table 4.

**Table 4:**
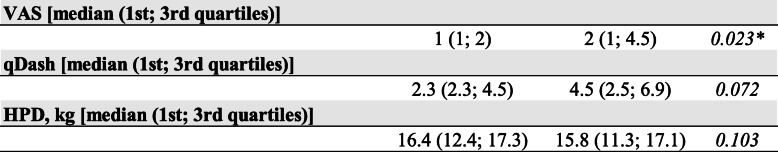
Functional outcomes six months postoperatively

### Complications

All k-wire and needles were removed after bone union. We did not burythe fixation devices, but the tubes of the needles were closed with a needle holder (Mayo-hegar type). No cases of infection were reported but five cases of delayed union occurred, one in group A and four in group B.

## Discussion

DP fractures recurring in young patients andwithout adequate treatment may lead to persistent fingertip pain and pinch dysfunction. Restoration of soft tissue is crucial in these lesions, and in cases of conservative treatment, some cases still required surgical fixation to facilitate bone union, maintain correct positioning to complete the healing process. In literature, surgery is indicated for unstable, displaced DP fractures [11] and in fracture with gap of fragments [12].

Due to size constraints, k-wire fixation is commonly used for management of DP fractures and nonunions. Screw fixation is an alternative and efficient technique in specific cases of displaced transverse DP shaft fractures [9, 10].

Some authors also described locked extra-articular pinning of the DIP joint, to reduced complications associated with current extra-articular and intra-articular techniques because the joint is not breached. Therefore, external connection of the two k-wires improves the stability of fixation [13].

We reported our intervention regarding fixation with two crossed hand drilled 23-G hypodermic needles. Besides Chen and Schneider [2], in literature one more needle fixation is described by Rha et al. [14] using a single 18-G hypodermic needlefor mallet finger with intra-articular fracture involving more than one-third of the articular surface.

23-G hypodermic needle are hollow and have a diameter of 0.6 mm. Commonly, longitudinal wires are used in DP fractures, in this study we compared both crossed needles and k-wire. Moreover, it is noteworthy to mention the crossed wire potential to provide stability in these fractures.In fact, Wang et al. [15] in a recent paper notedthe importance of nail restoration to biomechanical stability and a higher stability provided bytwo crossed k-wire.

Our results showed that the treatment with the two crossed drilled 23 G needle may lead to more efficient outcomesin terms of time to union in tuft and shaft fractures (Fig. 1, Tab. 2), with no significant differences in articular fractures.

Moreover, in group A, therange of movement at 6 months after trauma was higher (60°) compared to group B(40°).

Recent literature has reported that infections occurred more frequently in exposed k-wire cases than in buried k-wire ones [16] but we did not report any case of infections. We observed, however fivecases only of delayed union, most likelydue to the high comminution of the fracture.

We hypothesize that the most rapidbone union in group A compared to group B may be due to the needles’ diameter and the hole. This condition may result in betterbone vascularization after fracture and may facilitate a more rapid bone union. Further investigations are required to confirm this hypothesis.

Furthermore, hand-drilled needles allowed better surgeons’ control in needle orientation and permitted a better reduction and synthesis of small bone fragments from the fingertip. Moreover, this technique was performed in a surgery room ofour emergency department thus reducing healthcare costs and ensuring beneficial outcomes for the patients.

### Limitation of the study

We had tooutline that this study had some limitations related to the fact it is retrospective,and to the small sample size. Moreover, we can affirm that there could be a lack of randomization because the specific treatment was established by chance, that is the availability of operatory room in our hospital.

## Conclusion

In conclusion, this technique maybe performed in all clinical settings with safe, cost-effective and a satisfactory recovery process especially in tuft and shaft DP fractures. However, patients’ compliance and cooperation on fundamental requirements such as early mobilization is imperative to achieve an optimal outcome.

## Data Availability

The datasets generated during and/or analysed during the current study are available from the corresponding author upon reasonable request.

## Abbreviations

DP: Distal phalanx
k-wire: Kirschner-wires
ROM: Range of motion
ED: Emergency department
DIP: Distal interphalangeal
